# Assessing Root Causes of First Case On-time Start (FCOTS) Delay in the Orthopedic Department at a Busy Level II Community Teaching Hospital

**DOI:** 10.51894/001c.36719

**Published:** 2022-09-06

**Authors:** Blake Saul, Elise Ketelaar, Amjad Yaish, Michael Wagner, Robert Comrie, Grace D. Brannan, Carolina Restini, Michelle Balancio

**Affiliations:** 1 Orthopedic Trauma Surgeon Oschner Lafayette General Medical Center; 2 College of Osteopathic Medicine OMS-III Michigan State University (MSU); 3 Orthopedics Residency Program Director McLaren Macomb; 4 Orthopedics Residency Faculty McLaren Macomb; 5 Orthopedics PGY-5 Resident McLaren Macomb; 6 College of Osteopathic Medicine Michigan State University (MSU); 7 Orthopedics Department CRNA McLaren Macomb

**Keywords:** FCOTS, Quality Improvement, orthopedics, Ishikawa fishbone diagram, Pareto

## Abstract

**INTRODUCTION:**

Due to the high cost of operating room time, hospitals have been under increasing pressure to optimize operating room (OR) efficiency. One parameter that has been used to predict OR efficiency is First Case On-Time Start (FCOTS). In this brief report, the authors describe results from a quality improvement project designed to identify the rates and primary causes of first case delay for elective procedures within the orthopedic department at their suburban community hospital.

**METHODS:**

This was a retrospective, quality improvement project. The authors reviewed information from their anesthesia group to identify the rate and causes for delayed FCOTS, as well as observations and employee interviews to map contributing factors of delay.

**RESULTS:**

Surgery data on 159 days reviewed indicated that 107 (67.3%) days had first case delays. Of the 398 total first cases during this period, 156 (39.2%) were found to be delayed. The authors identified surgeon practices, with 74 (56.5%) as the main contributor to delay, followed by pre-operative processes, with 24 (18.3%), and room-related causes, 17 (13.0%). The anesthesia department and the patient were minor causes of delay, with 9 (6.9%) and 7 (5.3%) of case delays respectively.

**DISCUSSION:**

Results were similar to other studies, indicating surgeons and pre-operative as main cause for delay. A fishbone diagram revealed patient factors, inefficiency in the pre-operative process, and staff tardiness as some of the causes.

**CONCLUSIONS:**

During this project, surgeon practices and preoperative processes were the main factors contributing to OR inefficiency within the community-based hospital. Future strategies to improve daily OR flow within similar institutions should target surgeon on-time arrival and streamlining of the pre-operative process to effectively reduce FCOTS delays.

## INTRODUCTION

Operating room (OR) time is a very valuable and limited resource in the health care world. The average operating room costs approximately $21- $133 per minute, depending on factors such as the type of procedure with an average of $62 per minute.^1^ Therefore, improving OR utilization is critical to increasing the value of surgical services while decreasing overall healthcare costs.

First case on-time start (FCOTS), or the idea of starting the first operative cases scheduled for the day in each operating room on time, has long been used as an important OR metric due to its ability to predict inefficiency within an operative day.^2^ Delays in FCOTS leads to a ripple effect on subsequent cases throughout the day resulting in improper OR use, lower patient satisfaction, and increased costs within a hospital organization.^1^ Health care costs rise as OR rooms are staffed, but not able to be utilized.

Clinic-based procedures may require admission if later cases are scheduled for the following day.^3,4^ Less OR time also limits a surgeon’s total case volume which has financial implications. Although robust information is available regarding methods for improving FCOTS, there is limited published data identifying the most important factors leading to delay.^1–3^ Thus, it is beneficial for health care organizations to identify specific pre-operative (pre-op) causes of delayed FCOTS and determine effective methods for improving this metric to better serve their patient population, utilize time, and optimize revenue.

Several studies have attempted to determine causes of OR inefficiency.^2,5–7^ However, little literature is available which targets and quantifies the major factors contributing to first case delay. Identifying the main drivers of delay in FCOTS could lead to better implementation of strategies for improving this metric.^8^

At the authors’ community-based institution, the anesthesia team collected data concerning OR utilization and cause of delay, although these data nor the reasons for these delays had not been formally mapped. This brief report describes a quality improvement project conducted to identify the pre-op causes of FCOTS delay and determine effective methods for improving this metric to benefit the selected healthcare organization and patient population through efficient utilization of OR time and optimization of revenue.

## METHODS

### Project Design

This retrospective quality Improvement study was deemed as non-human subjects research by the McLaren System IRB and approved by our ethics committee (SARC 202004-01R1).

### Data Collection Procedures

The authors reviewed retrospective information from the anesthesia group at McLaren Macomb Hospital on causes for late starts in the orthopedic surgery department between April 1^st^, 2019, and December 31^st^, 2019, a time period with the most complete data. Data from elective cases occurring on Monday through Friday were collected to ensure full staffing was available. Days that had no operations were excluded from the total count. Additional data for the following variables were collected: number of elective surgery days, the total number of first cases for each day based on the number of ORs used, the instance of first case delays in any of the ORs, and the number of first cases delayed per day. In addition, the authors also collected the main reason for preoperative delay on each day based on one of five following reasons which were grounded in literature and our experience: surgeon, anesthesia, room, pre-op, or patient.^5–7^ Multiple reasons could be cited per day.

To complement the numerical data, the authors used observation and unstructured employee interviews to understand aspects of the admission process for an electively scheduled surgery and possible sources of delay. Employee interviews included the certified registered nurse anesthetist (CRNA) manager on staff, and multiple pre-op and post-op nurses. Employees were asked to explain the general “flow” of a patient’s encounter on their elective surgery day. No formal evaluation or selection process was used. This process was guided by an orthopedic resident (first author BS) during their five years within the community-based hospital system.

### Data Analysis

Author GB performed statistical analysis on categorical data. The authors generated the frequencies and percentages of cause of delay for our elective orthopedic first cases between Monday and Friday for a total of eight months. Chi-Square analyses was performed using IBM SPSS Statistics for Windows, Version 19.0 (Armonk, NY: IBM Corp). Statistical significance was set at a p value of < 0.05.

To further understand the impact of the reasons and sources of delays, two quality improvement tools were used. We used a Pareto chart, a bar graph with cumulative percentages, to visualize the most impactful causes of delay.^9^ We used Ishikawa’s fishbone diagram to map and illustrate the causes of delays and contributing factors. The diagrams were created using LucidChart Software (South Jordan, UT).

## RESULTS

**Figure 1.**
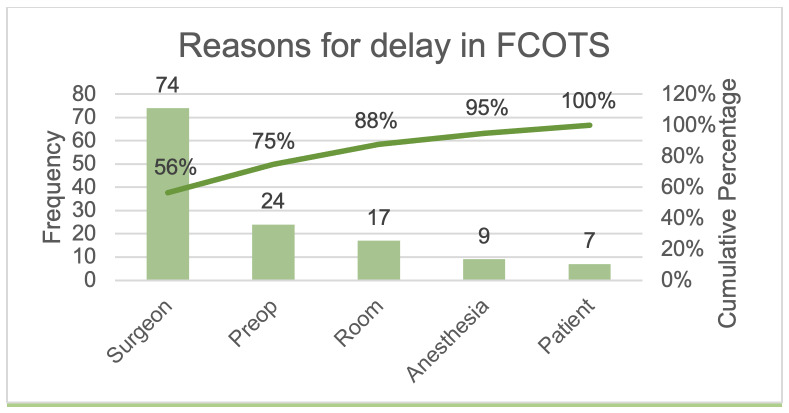
Pareto chart depicting reasons for delay in first case on-time start. [FCOTS)

A total of 159 days of elective orthopedic surgeries occurring on Monday through Friday were reviewed between the months of April 1^st^, 2019, and December 31^st^, 2019. Of these, 107 (67.3%) days had first case delays (p = 0.001). There were 398 first cases recorded over these 159 days. A total of 156 (39.2%) out of the 398 first cases were found to be delayed. The most significant causes of delay were found to be related to the surgeon, in 74 (56.5%) (e.g., late arrival to OR) instances, pre-op, 24 (18.3%), and room-related, 17 (13.0%) as depicted in Table 1. Anesthesia, 9 (6.9%) and patient, 7 (5.3%) were the least regular contributors to delay. Figure 1 depicts the Pareto chart for this data. Surgeon and pre-op combined consisted of 75% of the total causes of delay.

**Table 1.** Reasons for delay in first case on-time start.

**Reasons for delay**						
	Surgery	Pre-op	Room	Anesthesia	Patient	Total
Frequency	74	24	17	9	7	131
Percentage	56.5	18.3	13.0	6.9	5.3	100.0

**Figure 2.**
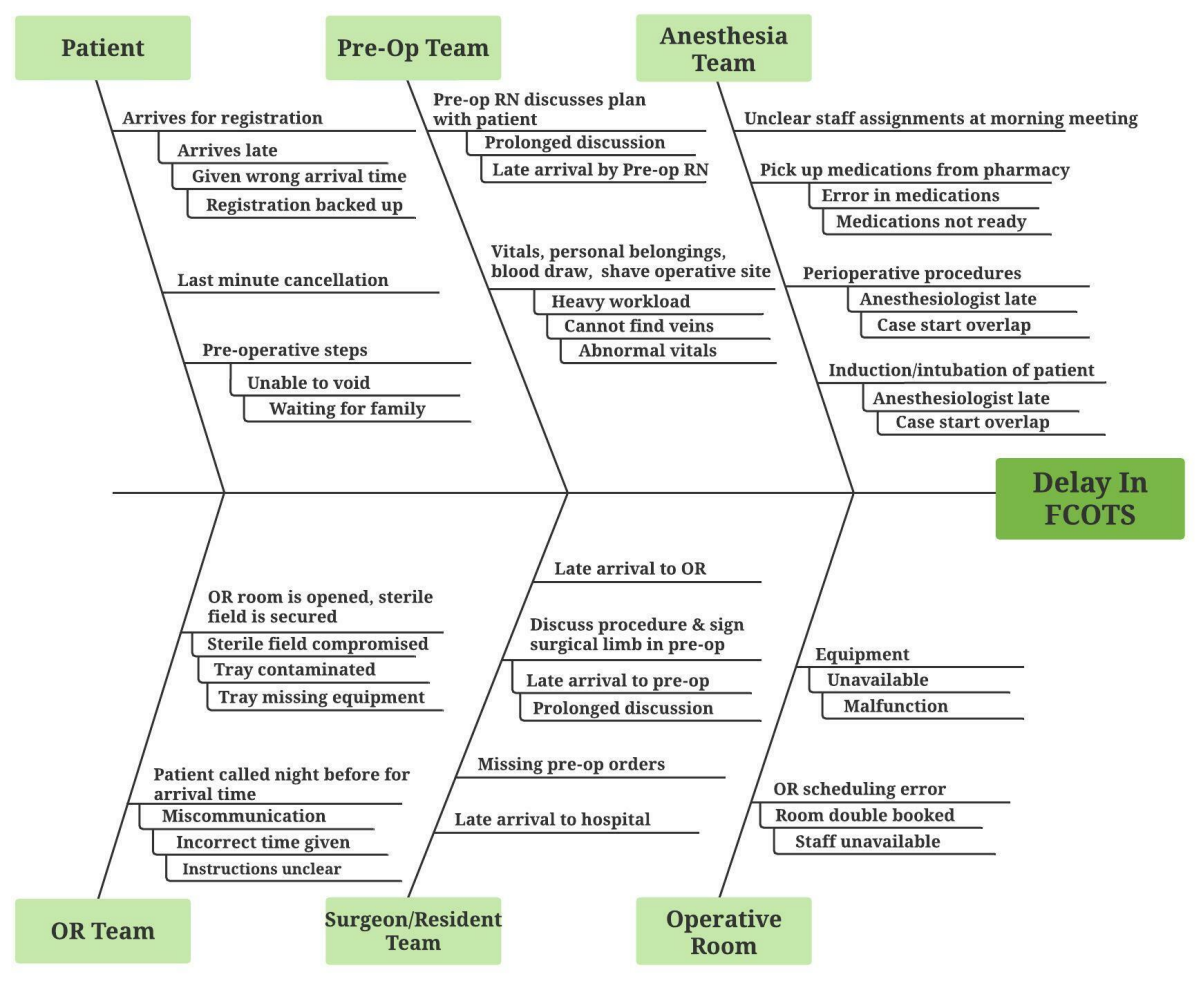
Cause and effect diagram depicting primary reasons for FCOTS delay and contributing factors. (Operative room [OR]), (First case on-time start [FCOTS]).

Many of the contributing factors were identified as depicted in Figure 2. Cause and effect tools like Ishikawa’s fishbone diagram allow analysts to identify primary factors and contributing factors to a problem. In this study some of the contributing factors affecting the five primary reasons for delays include a surgeon’s late arrival to the hospital or to the OR, missing or incorrect pre-op orders, difficulty obtaining vitals pre-op, pre-op nursing staff shortages, OR scheduling errors and missing equipment or equipment malfunction.

## DISCUSSION

Our institution’s average first case on-time start was 242 (60.8%), which, while comparable to an institutional median of 64.8%, is still below this reference value as reported in a 2012 study by the OR Benchmark Collaborative (ORBC).^9^ Compared to the 90^th^ percentile median of 88.3%, there is clearly room for improvement at our hospital.^10^ However, readers should consider that our data were collected solely from the orthopedic department, which could affect its external validity.

We identified surgeons’ practices as the leading cause of delay within our orthopedic department. While beyond the scope of this study, future research could explore all the contributing factors to prevent delays. Surprisingly, patients, in 7 (5.3%) instances, and anesthesia in 9 (6.9%) instances, were relatively minor sources of delay. The Pareto analysis (Figure 1) indicated that focusing on strategies to improve factors surrounding the surgeons and pre-op will have the biggest impact as these will address 75% of the challenges.

To our knowledge there were a few studies within similar institutions having identified surgeons as the main factor leading to delayed FCOTS.^1,6^ In 2016, Cox Bauer et al., retrospectively evaluated non-emergent first surgical case of the day from a few high-volume urban hospitals within the same health care system.^6^ The group found that out of 5,598 total cases 4,927 (88.01%) were delayed.^6^ Only 1,970 (40%) of delayed cases had documentation. Of these documented cases, the authors reported that physician was the most common reason for delay, in 1,024 (52%) instances. However, the reasons related to physicians was a limitation of the study as more specifics were not documented.

Other primary causes included anesthesia, 296 (15%) and patient factors (e.g. arriving late), 256 (13%).^6^ Although our study showed similar results, the data this 2016 group obtained from a wide range of surgical specialties included both elective and non-elective surgeries.^6^ Pashankar et al., also identified surgeons slow time in seeing patients in the pre op area at a tertiary care children’s hospital as a major contributor to delay in FCOTS over 12 months (24%).^1^ Around 27% of delayed cases had no documented reason for delay.^1^ Several other studies have identified surgeon arrival time as a major predictor of OR productivity.^11^

Although there is a general lack of information regarding specific causes of FCOTS, much published literature has explored strategies for improvement.^1,5,11–14^ Halim et al., reviewed 14 studies assessing the effectiveness of strategies to improve operating start time, finding the ‘golden patient’ technique i.e., (identifying the first patient on the next day’s OR list, and assuring they are medically optimized, and have already been seen by anesthesia) technique to save the most time.^12^ However, this 2018 study group specified that there is no “one-size-fits-all” approach, and each hospital system should identify specific causes of delay and tailor methods accordingly.^12^

Strategies aimed at surgeon preparedness such as financial incentive or communication, may be most applicable. For instance, a 2017 study group evaluated the impact of a pre-OR timeout and performance pay incentive on FCOTS.^13^ Attending surgeons with over 90% FCOTS over one year were qualified for a bonus of $1000 to $2000.^13^ This method improved the first case on-time starts by 57% over seven years and saved an estimated $751,120.^13^

### Project Limitations

First, while broad categories of delay were selected based on experience and the literature, it could have limited our ability to include other possible causes of FCOTS delay. Future studies should further investigate information on other potential primary causes, such as causes for patient delay (e.g., late arrival to the hospital, improper documentation, lack of surgical clearance, non-adherence to preoperative fasting restrictions).

Second, our current data collection protocol provided the OR nurse or CRNA with full discretion to list the reason for delay which leaves room for potential bias. Third, this was a retrospective project with manual data collection that could have skewed final results. In the future, our institution plans to refine perioperative protocols to address FCOTS delay factors and improve patient satisfaction rates within the orthopedic service.

## CONCLUSION

Our retrospective quality project with institutional data indicated that surgeons’ practices and inefficient pre-op processes were the main contributors to OR inefficiencies. Future targeted strategies to improve daily OR flow within community-based institutions should focus on surgeon arrival times and streamlining of the pre-op process.

### Conflict of Interest

None
